# Dose and timing of injections for effective cyclosporine A pretreatment before renal ischemia reperfusion in mice

**DOI:** 10.1371/journal.pone.0182358

**Published:** 2017-08-10

**Authors:** Sandrine Lemoine, Bruno Pillot, Lionel Augeul, Maud Rabeyrin, Annie Varennes, Gabrielle Normand, Delphine Baetz, Michel Ovize, Laurent Juillard

**Affiliations:** 1 Université Lyon1, Inserm 1060CarMeN, Lyon, France; 2 Renal function unit, Edouard Herriot Hospital, Hospices Civils de Lyon, Lyon, France; 3 Nephrology department, Edouard Herriot Hospital, Hospices Civils de Lyon, Lyon, France; 4 Anatomopathology, Edouard Herriot Hospital, Hospices Civils de Lyon, Lyon, France; 5 Biology department, Edouard Herriot Hospital, Hospices Civils de Lyon, Lyon, France; 6 Cardiovascular Explorations, Hospices Civils de Lyon, Louis Pradel Hospital, Lyon, France; University of PECS Medical School, HUNGARY

## Abstract

**Background:**

There is experimental evidence that lethal ischemia-reperfusion injury (IRI) is largely due to mitochondrial permeability transition pore (mPTP) opening, which can be prevented by cyclosporine A (CsA). The aim of our study is to show that a higher dose of CsA (10 mg/kg) injected just before ischemia or a lower dose of CsA (3 mg/kg) injected further in advance of ischemia (1 h) protects the kidneys and improves mitochondrial function.

**Methods:**

All mice underwent a right unilateral nephrectomy followed by 30 min clamping of the left renal artery. Mice in the control group did not receive any pharmacological treatment. Mice in the three groups treated by CsA were injected at different times and with different doses, namely 3 mg/kg 1 h or 10 min before ischemia or 10 mg/kg 10 min before ischemia. After 24 h of reperfusion, the plasma creatinine level were measured, the histological score was assessed and mitochondria were isolated to calculate the calcium retention capacity (CRC) and level of oxidative phosphorylation.

**Results:**

Mortality and renal function was significantly higher in the CsA 10 mg/kg-10 min and CsA 3mg/kg-1 h groups than in the CsA 3mg/kg-10 min group. Likewise, the CRC was significantly higher in the former two groups than in the latter, suggesting that the improved renal function was due to a longer delay in the opening of the mPTP. Oxidative phosphorylation levels were also higher 24 h after reperfusion in the protected groups.

**Conclusions:**

Our results suggest that the protection afforded by CsA is likely limited by its availability. The dose and timing of the injections are therefore crucial to ensure that the treatment is effective, but these findings may prove challenging to apply in practice.

## Introduction

Acute kidney injury (AKI) can lead to chronic kidney disease, requiring renal replacement therapy, but can also cause cardiovascular events and death. The increasing number of patients developing AKI is therefore a significant public health concern[1]. Even in young people, AKI is associated with increased use of renal-replacement therapy [2]. Moreover, although it is often reversible, AKI significantly increases the risk of de novo chronic kidney disease [3]. Since ischemia-reperfusion injury (IRI) is the most common cause of AKI, there is considerable interest in developing pharmacological treatments to protect the kidneys from IRI, particularly when this is predictable.

It is now widely accepted that mitochondrial dysfunction, especially the opening of mitochondrial permeability transition pore (mPTP), plays a major role in aggravating the lesions following cardiac IRI. This opening causes the mitochondria to swell and the membrane potential to collapse, and uncouples oxidative phosphorylation. The opening of the mPTP is facilitated by the binding of a protein, cyclophilin D (CyP-D), to the inner membrane of the mitochondria. Previous heart studies have demonstrated that cyclosporine A (CsA). independently of its anti-calcineurin properties, protects from IRI by binding CyP, thus preventing mPTP opening [4,5].

We have shown previously that CsA injection at the reperfusion stage (postconditioning) may protect the kidneys from IRI by inhibiting mPTP opening [6]. Cyclosporine A can only be injected after reperfusion following acute myocardic or brain infarction; in nephrology however, the clinical situations (artery stenosis revascularization, transplantation, aortic clamping for vascular surgery) are such that reperfusion is often programmed, allowing CsA to be administered before ischemia. In the only previous studies of CsA injections to preserve renal function, CsA was administered 6 h before kidney ischemia [7–9]. This timing was chosen based on the hypothesis that CsA increases the activity of the heat shock protein system (HSP70). In clinical practice however, injecting CsA such a long time (several hours) before ischemia may not be possible. Our hypothesis is that CsA protects renal function because CsA inhibits mPTP opening, justifying the use of CsA just before ischemia. Moreover, whereas the CsA dose used in previous renal studies was 3 mg/kg, in the heart or brain, 10 mg/kg was used [10,11]. The dose and timing of CsA injections prior to renal ischemia reperfusion are parameters that are both worth optimizing in this context.

The aim of our study is to investigate whether a high dose of CsA (10 mg/kg) injected just before ischemia or a lower dose of CsA (3 mg/kg) injected 1 h before ischemia protects the kidneys and improves mitochondrial function. We therefore compared the effects on renal and mitochondrial function of two different doses of CsA (3 or 10 mg/kg) injected either 10 min or 1 h before ischemia.

## Materials and methods

### Surgical preparation

The animals were maintained in a controlled environment, in compliance with European recommendations on laboratory animal care and the protocols were approved by the Ethics Committee of the Université Claude Bernard Lyon 1 (approval number: BH2012-81).

Male C57BL/6J mice, aged 8–12 weeks and weighing 25–30 g were purchased from approved suppliers (Charles River Laboratories, France). The mice were raised with free access to food (SAFE, France) and water in a 12 h light/dark cycle.

The mice were anesthetized by intraperitoneal injection of xylazine (5 mg/kg, Rompun^®^, Bayer, Puteaux, France), ketamine (100 mg/kg, Imalgene^®^ 1000, Acyon, Melan, France), and bupremorphine (0,075mg/kg, Vetergesic^®^, Sogeval, Laval, France). Their body temperature was monitored and maintained at 37°C using a homeothermic pallet unit.

The animals were intubated orally using a 22-gauge catheter and ventilated with a rodent ventilator (Minivent, Harvard Apparatus, March, Germany) with a volume of 0.15 mL and a breath rate of 140 /min. After laparotomy and right nephrectomy, a 30 min period of ischemia of the left kidney was imposed by clamping the left renal vascular pedicle using a microvascular clamp (Roboz Surgical Instruments, Washington, DC). Ischemia and reperfusion were confirmed visually by observing the color of the left kidney.

After surgery, roughly every 3 h during daytime (three to four times a day), the mice were weighed and their welfare was assessed using Langford et al.’s pain scale [12]. Mice who suffered more than 20% weight loss or had a pain score higher than 2, were given a 0.1 mg/kg subcutaneous injection of buprenorphine. If their health had not improved 3 h after analgesia, the animals were euthanized on the advice of the veterinary advice.

### Study design

The two doses of CsA (3 and 10 mg/kg) were first tested in sham mice (four in both groups), 24 h after reperfusion, to rule out acute nephrotoxicity. All measures in sham mice were made after 24hrs of reperfusion.

The untreated group named “control group” (n = 8) was then compared with three different preconditioned groups: the first injected with 3 mg/kg CsA 1 h before ischemia (CsA 3 mg/kg-1 h; n = 6), the second injected with 3 mg/kg CsA 10 min before ischemia (CsA 3 mg/kg-10 min; n = 6), and the third injected with 10 mg/kg CsA 10 min before ischemia (CsA 10 mg/kg-10 min; n = 6). The vehicle used in the control group was 0.9% saline. The same volumes were injected in all mice to ensure that the hemodynamic effects were identical.

All animals (i.e. from the sham groups, control group and the three preconditioned groups) underwent right nephrectomy followed by a 24 h reperfusion period. Immediately after the surgical procedure, the mice were hydrated by injecting 1 mL of 0.9% saline subcutaneously. After 24 h of reperfusion, deep anesthesia was induced once more (using ketamine and xylazine), 250 μL of blood was extracted by retro-orbital puncture, and the kidney was rapidly removed and divided into two samples, one to isolate mitochondria and one for histological analysis. The mice were then euthanized by cervical dislocation.

### Renal parameters

Acute renal failure was assessed 24 h after reperfusion by measuring the serum concentration of creatinine. Serum creatinine was measured enzymatically (Vista 1500, Siemens Healthcare, Erlangen, Germany). The renal tissue was embedded in formaldehyde. Four-micrometer sections were stained with periodic acid-Schiff reagents. Tubular injury was scored semi-quantitatively from zero to four by a blinded pathologist who examined at least 10 fields at ×200 magnification. Tubular injury was defined as a tubular dilatation, sloughing of tubular epithelial cells, or a naked tubular basement membrane. Only tubules in the outer stripe of the outer medulla (the most sensitive zone for ischemic injury) were included in the following scoring system: 0, no tubular injury; 1, < 20% of damaged tubules; 2, 21–50% of damaged tubules; 3, > 51% of damaged tubules; 4, total destruction of all epithelial cells [6].

### Mitochondrial function

#### Preparation of isolated mitochondria

The kidneys were excised and immediately placed in a cold isolation buffer (0.25 M sucrose, 2 mM EGTA and 10 mM Tris, pH 7.4). The tissue was cut with scissors and mixed in the same buffer (10 μL buffer/mg tissue) with a Potter-Elvehjem homogenizer. The homogenate was centrifuged at 600 g for 7 min and the supernatant at 6400 g for 10 min. The mitochondrial pellet was suspended and homogenized in the same buffer without a calcium chelating agent. The protein concentration was determined using the Bradford method.

#### Calcium retention capacity

The calcium retention capacity (CRC) is the amount of Ca^2+^ required to trigger massive Ca2+ release (by the mitochondria into the cytosol as described by Ichas et al. [13]). The CRC quantifies the resistance to opening of the mPTP following a calcium overload. Isolated mitochondria (250 μg protein) were suspended in 2 mL of buffer C (150 mM sucrose, 50 mM KCl, 2 mM KH2PO4, 5 mM succinic acid in 20 mM Tris–Hcl pH 7.4) in a polystyrene cuvette. The extra-mitochondrial Ca2+ concentration was measured with a Hitachi F2500 spectrofluorometer in the presence of 0.8 μmoL Calcium Green-5N (with excitation and emission wavelengths set at 500 and 530 nm, respectively). CaCl_2_ (10 nmol) was injected every minute. Each CaCl2 pulse in the cuvette increases the extra-mitochondrial Ca^2+^ concentration. The Ca^2+^ is then absorbed by the mitochondria, such that the extra-mitochondrial Ca^2+^ concentration decreases rapidly. After several additions of extra-mitochondrial calcium, an abrupt increase in the extra-mitochondrial Ca^2+^ concentration indicates a massive release of Ca^2+^ by the mitochondria due to mPTP opening. The total amount of calcium required to open the mPTP was calculated and expressed in nmol Ca^2+^/mg mitochondrial protein.

Secondly, we added 10 μL of CsA into the cuvette directly in contact to mitochondria.

#### Oxidative phosphorylation

Mitochondrial respiratory function was measured at 25°C using a Clark-type electrode (Oroboros oxygraph, Austria). Mitochondria (250 μg protein) were incubated in a respiration buffer (pH 7.4) containing 80 mM KCl, 50 mM MOPS, 1 mM EGTA, 5 mM KH2PO4, 1mg/mL fat free BSA and 3 mM each of pyruvate, malate and glutamate (complex I substrate). Oxygen consumption was expressed in nmol O_2_/min/mg of protein. Oxygen consumption is maximal in the actively respiring state, state 3, as stimulated by ADP when all the required components are present. The quiescent state, state 4, corresponds to the lower oxygen consumption after ADP phosphorylation. The respiratory control index (RCI) is the ratio of oxygen consumption rates in these two states (state 3/state 4) and indicates the tightness of the mitochondrial coupling between respiration and phosphorylation.

### Statistical analysis

In the following, results are expressed as the mean ± the SEM. The difference between the sham groups was assessed by one-way ANOVA with a Bonferroni correction. Each preconditioned group was compared with the control group using Mann-Whitney tests (Graphpad software^®^). CsA concentrations were also compared using Mann-Whitney tests. The threshold for statistical significance was p < 0.05.

## Results

### Effect of CsA on kidney function in sham mice

**Fig 1:**
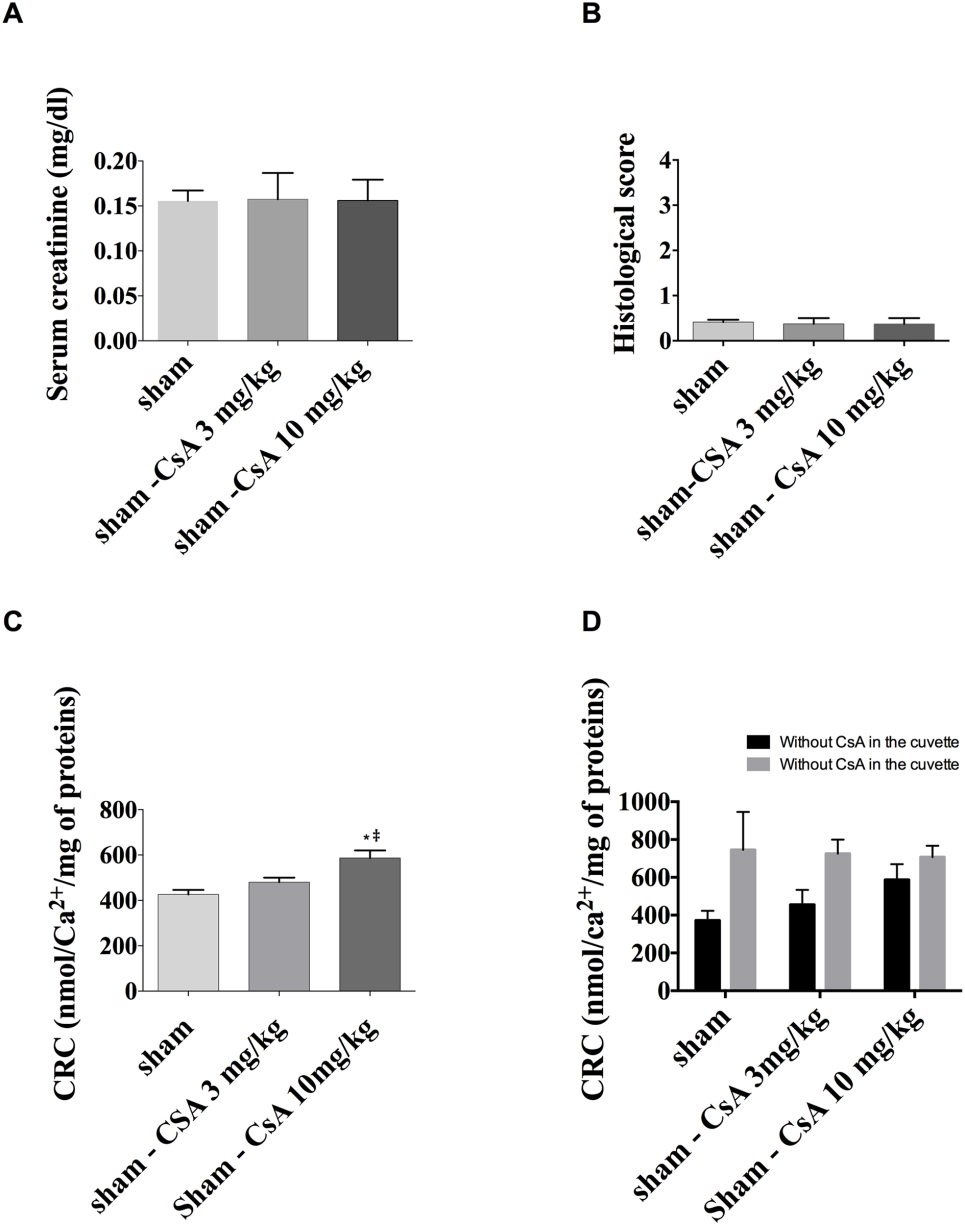
(A) Serum creatinine levels, (B) histological scores and (C) calcium retention capacity of CsA-treated and untreated sham mice after 24 h of reperfusion following renal ischemia. The three groups only differ in terms of their calcium retention capacity, which is significantly higher in the group that was given 10 mg/kg CsA than in the mice that were given a 3 mg/kg dose. D. Effect of addition of CsA directly into the cuvette on Calcium Retention capacity. Addition of CsA directly on mitochondria increased CRC in all groups.

There is no difference between the three groups of sham mice in terms of serum creatinine concentrations (0.13, 0.13 and 0.13 mg/dL, for the sham, sham 3 mg/kg and sham CsA 10 mg/kg groups respectively, p = 0.9) or in terms of the histology score (p = 0.9 between the groups) (Fig 1A & 1B).

The residual CsA concentration 24 h after injection was 30 and 36 μg/L for the sham CsA 3 mg/kg and sham CsA 10 mg/kg mice, respectively, while the mean arterial blood pressure (MAP) in these two groups was 103 ± 24 and 119 ± 22 mmHg, respectively, but without a hypertensive peak.

The CRC of the sham mice treated with 10 mg/kg CsA before ischemia was significantly higher than that of the untreated mice or the group treated with 3 mg/kg CsA (586 ± 82 versus 426 ± 50 and 480 ± 50 nmol Ca2+/mg protein respectively; p<0.001) (Fig 1C). Moreover, after the addition of 10 μL of CsA into the cuvette, CRC was significantly increased, and up to a similar level, in all groups (Fig 1D).

### Effect of CsA on mice survival

The mortality after 24 h of reperfusion was significantly higher in the control (untreated) group and CsA 3 mg/kg-10 min groups than in the sham group (40 and 41% respectively, p<0.01). The mortality rates in the control group and CsA 3 mg/kg-10 min groups were not significantly different (p = 0.2). The mortality rate after 24 h reperfusion was significantly lower in the CsA 3 mg/kg-1 h group (10%, p<0.05) and in the CsA 10 mg/kg-10 min group (0%, p<0.05) than in the control group.

### Optimal CsA dose for renal protection

**Fig 2:**
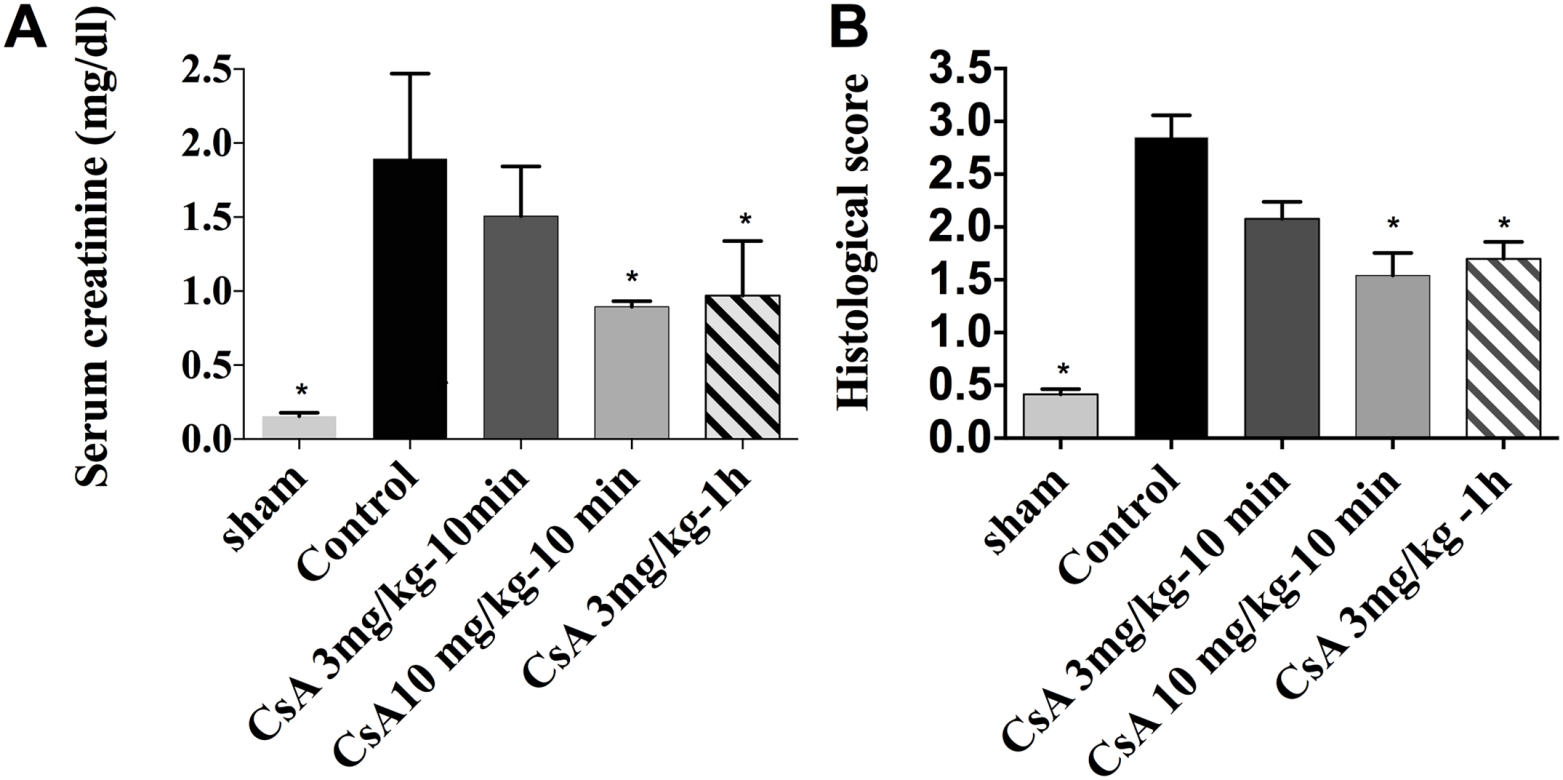
Impact of the dose of CsA on renal function. (A) Plasma creatinine levels, (B) histological scores 24 h after reperfusion (*p< 0.05 vs control).

The creatinine concentration in the CsA3 mg/kg-10 min group (1.5 ± 0.3 mg/dL) was no different from that of the control group (1.9 ± 0.6 mg/dL, p = 0.4), but the corresponding levels in the CsA 10 mg/kg-10 min and CsA 3 mg/kg-1 h groups were significantly lower (0.96 ± 0.36 and 0.9 ± 0.33, p = 0.0007 and p = 0.001, respectively; Fig 2A). The histological scores for the latter two groups were likewise lower than in the control group (1.5 ± 0.5, p = 0.002 and 1.7 ± 0.3, p = 0.004 respectively, versus 2.85 ± 0.5), but the difference between the histological scores of the control and CsA 3 mg/kg-10 min groups is not significant (2 ± 0.3 for the latter, p = 0.2) (Fig 2B).

### Effect of CsA on kidney mitochondria function

**Fig 3:**
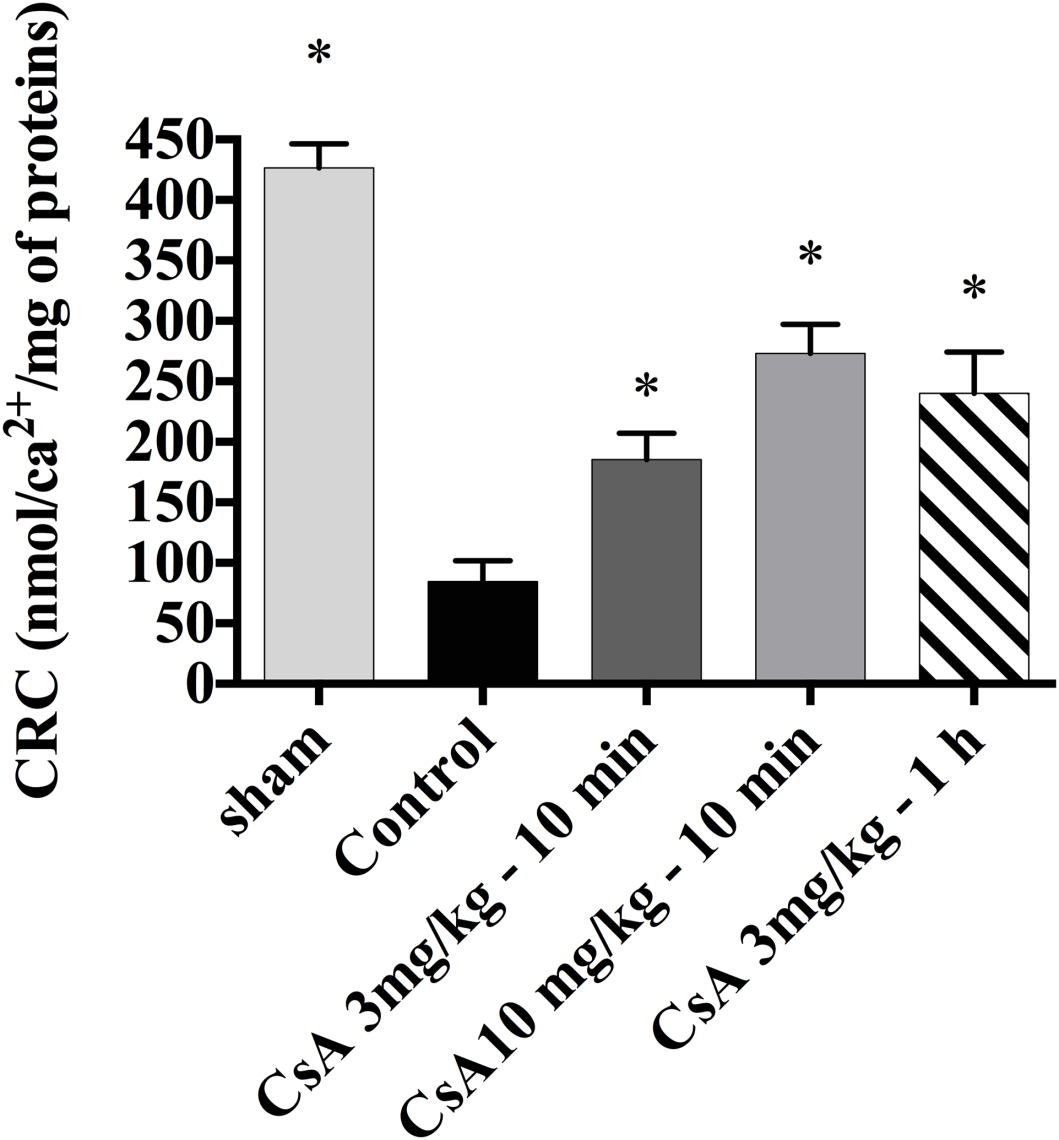
Impact of the dose of CsA on mitochondrial function. (A) Calcium retention capacity (CRC) 24 h after reperfusion as a function of the CsA dose. Mitochondrial PTP opening was significantly inhibited in the control versus the sham group. The CRC 24 h after reperfusion was significantly higher in the CsA 10 mg/kg-10 min group than in the control or CsA 3 mg/kg-10 min groups (*p< 0.05 vs control group).

After 24 h of reperfusion, the mitochondrial CRC was dramatically lower in the control group than in the sham group (84 ± 5 versus 426 ± 48 nmol Ca2+/mg protein, p = 0.001). The CRC after 24 h reperfusion for the CsA 3 mg/kg-10 min, CsA 10 mg/kg-10 min and CsA 3 mg/kg-1 h groups (respectively 185 ± 53, 273 ± 58, and 228 ± 82 nmol Ca2+/mg protein) were significantly higher (p<0.001) than for the control group (84 ± 5 nmol Ca2+/mg protein; Fig 3).

After 24 h of reperfusion, state 3 oxygen consumption was much lower in the control group than in the sham group, (**27** ± 15 nmol O_2_/min/mg protein), but was higher in the CsA 3 mg/kg-10 min, CsA 10 mg/kg-10 min and CsA 3 mg/kg-1 h groups than in the control group (54 ± 20, 45 ± 7 and 40 ± 8 versus 27 ±15 nmol O_2_/min/mg, p = 0.01, 0.04 and 0.04 respectively, see Table 1). The RCI of the CsA 10 mg/kg-10 min (3.9 ± 0.3) and CsA 3 mg/kg-1 h (4.1 ± 0.36) groups was significantly higher than that of the control group (3.9 ± 0.3 and 4.1 ± 0.36 versus 2 ± 1, p = 0.004 and 0.008 respectively), but the RCI of the CsA 3 mg/kg-10 min group (3 ± 0.9) was not significantly different from the control group’s (p = 0.6). In other words, the rate of oxidative phosphorylation was lower in the CsA 3 mg/kg-10 min group than CsA 3mg/kg-1h and CsA 10 mg/kg-10 min.

**Table 1:** Oxidative phosphorylation 24 h after reperfusion in CsA-treated, sham and control mice.

	State 3 nmol O_2_/min/mg of protein	State 4 nmol O_2_/min/mg of protein	RCI
Sham	63 ± 15 *	12 ± 2 *	5.2 ± 0.7 *
Control group	27 ± 15	10 ± 6	2 ± 1
CsA 3 mg/kg-10 min	54 ± **20** *	16 ± 3	3 ± 0.9
CsA10mg/kg-10 min	4**5** ± 7*	10 ± 2	3.9 ± 0.3 *
CsA 3 mg/kg-1 h	40 ± 8 *	9.8 ± 2.7	4.1 ± 0.3*

State 3 and state 4 were measured in isolated mitochondria with pyruvate/malate/glutamate as substrates of complexe I and are expressed in nmol O_2_/min/mg of proteins. RCI: respiratory control index (state3/state4). Values shown are the mean ± SD. (n = 4 to 6).

* p< 0.05 vs control group using a Mann Whitney test.

## Discussion

This study shows that a single dose of CsA immediately before ischemia improves renal function and morphology. The results also indicate that a lower dose of CsA is also effective if it is injected 1 h before ischemia. A plausible interpretation is that the renal protection afforded by CsA depends on the dose injected and on the timing of the injection. The positive effect of both these factors on renal function seems to stem from a delaying of mPTP opening and an improvement in oxidative phosphorylation.

### The impact of the CsA dose at the time of reperfusion

In renal ischemia-reperfusion, ischemic preconditioning [14] and postconditioning [15] have both been successfully tested in laboratory environments. While remote renal ischemic pre- and postconditioning have also been investigated in a clinical setting, no consistent effect has been reported [16]. Although pharmacological renal conditioning is easier to perform, our study of different doses and timings of CsA injections adds to a very sparse literature on this subject [7–9, 17]. A 3 mg/kg dose of CsA injected 10 min before ischemia did not improve renal function or the histological score of mice 24 h after reperfusion. The effects of CsA have been shown to be related to its concentration in various organs and settings. In the liver, Soriano *et al*. found that mPTP inhibition was better with a 5 mg/kg dose of CsA than with a dose greater than 10 mg/kg [18]. In the heart, Chen *et al*. have also suggested that the protective and cytotoxic effects of CsA might be dose-dependent [19]. In the brain, histological analyses three days after stroke revealed that only those ischemic animals treated with high dose CsA had significantly reduced cerebral infarcts [20] In the kidney however, this impact of the dose has only been demonstrated in a postconditioning study [21]. An explanation for previous studies with negative results may be that the timing of the injections was inappropriate and/or that the concentration of CsA upon reperfusion was too low [22–24]. For example, while Martinez-Palli *et al*. reported that CsA does not improve renal function [22], their injections were administered *per os* 24 and 6 h before transplantation at 5 mg/kg. The concentration of CsA at the time of ischemia was therefore only 197 ng/mL, lower than in our study. Delbridge *et al*. found no positive effect of CsA injected *per os* for 30 days after ischemia reperfusion (16). However, these injections occurred too late after reperfusion and the repetitive high dose was probably toxic. This heterogeneity of results is probably a consequence of the different doses and modes of CsA injection (*per os*, intravenous, subcutaneous). Our study suggests that the CsA concentration at the time of reperfusion is crucial to its protective effect on renal function. This would explain why a 10 mg/kg dose of CsA injected intravenously 10 min before ischemia offered renal protection while a lower dose (3 mg/kg) was only found to be protective if it was injected much longer before ischemia. The fact that 3mg/kg– 1hr lead to a significant protection whereas the 3mg/kg– 10min could be explain by the availability in the kidney of the drug. More we injected CsA, more CypD can be bound and more mPTP remains closed.

### Role of the mitochondria and mPTP in renal ischemia-reperfusion

Previous studies investigated the effects of CsA administered 6 h before ischemia because of the molecule’s presumed benefit through the overexpression of HSP70. The HSP70 system protects the mitochondria from reactive oxygen species by maintaining ATP levels, repairing DNA damage and preserving tight junction integrity. Importantly in this context moreover, it has been shown to prevent calcium redistribution within the cell in renal ischemia-reperfusion [25]. There is also experimental evidence that the mitochondria also play a crucial role in AKI. Mitochondria have a dual role in the kidney as the primary source of energy for tubular cells and as a key regulator of cell death via the opening of the mPTP. Our study demonstrates for the first time that pretreatment with CsA inhibits mPTP opening 24 h after renal ischemia-reperfusion in a dose-dependent way. It also shows that CsA improves mitochondrial respiration, which is a substantial benefit in renal ischemia. However, we didn’t find any modification of complex II activity with CsA treatment (data not shown).

However, the mPTP is not the only pathway to protect renal function. In a previous review, Zuk and Bonventre list the different pathophysiological mechanisms of AKI: renal vasculature and cellular stress responses, endothelium reticulum stress, mitochondrial dysfunction, autophagy, and innate immune response and maladaptive repair [26]. While investigating all these pathways was not feasible in our study, we propose a pharmacological approach to protect the kidneys against mitochondrial dysfunction in AKI.

### Toxicity of CsA

A 10 mg/kg dose of CsA is usually considered nephrotoxic. However, the fact that CsA was administered as a single bolus in this study reduces the risk of vascular toxicity. Indeed, although CsA increases renal vascular resistance and decreases renal blood flow, the hemodynamic impact of CsA is transient (<10 min) as has been demonstrated experimentally by other groups [27–28]. In this study also, the absence of any effect of CsA on the renal function and blood pressure of sham mice indicates that the single bolus administered is not nephrotoxic. No lesions were observed after CsA injection, even in the mice that received the 10 mg/kg dose.

In conclusion, our results suggest that CsA-induced protection against ischemia-reperfusion injury is dose-dependent and is likely mediated by the inhibition of mPTP opening, which would explain why the concentration of CsA has to be high at the time of reperfusion. In clinical practice therefore, the dose and the timing of CsA injections should be carefully managed in order to achieve the desired preconditioning effect.
